# Delayed Sequelae of Neonatal Respiratory Syncytial Virus Infection Are Dependent on Cells of the Innate Immune System

**DOI:** 10.1128/JVI.02620-13

**Published:** 2014-01

**Authors:** James A. Harker, Yuko Yamaguchi, Fiona J. Culley, John S. Tregoning, Peter J. M. Openshaw

**Affiliations:** Department of Respiratory Medicine, Centre for Respiratory Infection, National Heart and Lung Institute, Imperial College London, London, United Kingdom

## Abstract

Infection with respiratory syncytial virus (RSV) in neonatal mice leads to exacerbated disease if mice are reinfected with the same virus as adults. Both T cells and the host major histocompatibility complex genotype contribute to this phenomenon, but the part played by innate immunity has not been defined. Since macrophages and natural killer (NK) cells play key roles in regulating inflammation during RSV infection of adult mice, we studied the role of these cells in exacerbated inflammation following neonatal RSV sensitization/adult reinfection. Compared to mice undergoing primary infection as adults, neonatally sensitized mice showed enhanced airway fluid levels of interleukin-6 (IL-6), alpha interferon (IFN-α), CXCL1 (keratinocyte chemoattractant/KC), and tumor necrosis factor alpha (TNF-α) at 12 to 24 h after reinfection and IL-4, IL-5, IFN-γ, and CCL11 (eotaxin) at day 4 after reinfection. Weight loss during reinfection was accompanied by an initial influx of NK cells and granulocytes into the airways and lungs, followed by T cells. NK cell depletion during reinfection attenuated weight loss but did not alter T cell responses. Depletion of alveolar macrophages with inhaled clodronate liposomes reduced both NK and T cell numbers and attenuated weight loss. These findings indicate a hitherto unappreciated role for the innate immune response in governing the pathogenic recall responses to RSV infection.

## INTRODUCTION

Most cases of infantile viral bronchiolitis are caused by respiratory syncytial virus (RSV) infection (1). Infants previously hospitalized with RSV disease are highly likely to experience recurrent wheeze (2, 3), and delaying RSV infection (with the monoclonal antibody palivizumab) reduces the prevalence of wheezing during the first year of life (4). The mechanisms responsible for postbronchiolitic wheeze are not fully defined, but it is possible that severe viral lung infections during infancy cause long-term changes in the mucosal immune responses in the lung.

To investigate the long-term effects of viral lung infection in early life, we developed a mouse model of neonatal RSV infection followed by adult reinfection (5). Both CD4 and CD8 T cells play a role in this phenomenon, as does the major histocompatibility complex (MHC) genotype (6, 7), but these factors may not fully explain the delayed effects of neonatal sensitization, since T cell depletion during secondary reinfection does not completely abrogate disease. The presence of weight loss as early as day 2 after infection (6) suggests the possibility that innate responses also play a role.

When neonatal mice are infected with a recombinant RSV expressing interleukin-4 (IL-4), weight loss is not exacerbated during adult reinfection, despite greatly enhanced Th2 immunity (8). In addition, while neonatal infection with RSV expressing gamma interferon (IFN-γ) is protective against disease during subsequent reinfection, it does not enhance Th1 immunity; rather, the clearest correlate of protection is reduced natural killer (NK) cell recruitment during secondary reinfection. Since strong NK cell responses can cause severe pathology during adult RSV infection (9, 10), we wished to determine whether the innate response plays a role in orchestrating or enhancing the pathology seen during secondary reinfection in mice neonatally infected with RSV.

In the present studies, we found that RSV infection during infancy (but not adulthood) led to a rapid increase of highly activated NK cells in the airways and lungs. Removal of NK cells or the alveolar macrophages necessary for their recruitment resulted in a reduction in subsequent disease. Thus, cells of the innate immune system play an important role in the long-term effects of neonatal RSV infection.

## MATERIALS AND METHODS

### Virus stocks and mouse infection.

RSV strain A2 was grown in HEp-2 cells, and the viral titer was determined by plaque assay. Time-mated pregnant BALB/c mice (Harlan, Motspur Park, United Kingdom) were purchased at <14 days of gestation, and pups were weaned at age 3 weeks. Mice were infected intranasally (i.n.) with 4 × 10^4^ focus-forming units (FFU)/g body weight virus at 4 days (neonatal dose was ∼10^5^ focus-forming units [FFU]) while they were under isoflurane anesthesia. Secondary RSV challenge was given i.n. at 10^6^ FFU in 100 μl at 8 weeks after priming. Weight was measured daily to monitor disease severity. All work was approved and licensed by the United Kingdom Home Office.

### Cell depletion.

To deplete NK cells, 100 μl of rabbit anti-mouse asialo-GM1 polyclonal antibodies (Wako Chemicals, Japan) or control antibodies was administered intravenously (i.v.) on days −1 and +2 of infection; to deplete basophils, anti-FcεR1 (MAR1) was administered intraperitoneally on days −1 and +2 of secondary reinfection. NK cell depletion was performed either during secondary reinfection or primary infection or between the primary and secondary infections. For macrophage depletion, mice were treated prior to secondary reinfection with 100 μl of a clodronate-containing liposome (CL) suspension (a gift from Nico van Rooijen, Boehringer GmbH, Germany) or the control treatment i.n. while the they were under light anesthesia with isoflurane.

### Quantification of viral RNA.

RNA was extracted from the lung using the RNA stat-60 reagent (AMS Biotech Ltd.), and cDNA was generated with random hexamers using Omniscript reverse transcriptase (Qiagen). Real-time PCR was carried out with a sequence in the RSV L gene using 900 nM forward primer (5′-GAACTCAGTGTAGGTAGAATGTTTGCA-3′), 300 nM reverse primer (5′-TTCAGCTATCATTTTCTCTGCCAAT-3′), and 100 nM probe (5′-FAM-TTTGAACCTGTCTGAACAT-TAMRA-3′, where FAM is 6-carboxyfluorescein and TAMRA is 6-carboxytetramethylrhodamine) on an ABI Prism 7000 sequence detection system. This detects viral genomic RNA, viral anti-genomic RNA, and intracellular RSV L mRNA, referred to here as RSV L RNA gene. The L-gene copy number was quantified relative to that for a standard plasmid (5).

### Cell recovery.

Collection of bronchoalveolar lavage (BAL) fluid for cells and supernatants and the harvesting of lung tissues were carried out as previously described (11). For the preparation of lung mash supernatants, lungs were homogenized through 100-μm-mesh-size cell strainers (BD Pharmingen) and washed through with a 1-ml volume of RPMI 1640 five times. Following centrifugation, this supernatant was retained for enzyme-linked immunosorbent assay (ELISA) analysis. After the removal of the supernatants from all tissues, cells were treated with ACK lysing buffer (150 mM ammonium chloride, 10 mM potassium bicarbonate, 0.1 mM EDTA) for 5 min and then DNase I (40 μg/ml)-collagenase (50 μg/ml) in RPMI 1640 (with 10% fetal calf serum) for 5 min at room temperature to remove clumps. Finally, they were resuspended in RPMI 1640. Cell viability was assessed by trypan blue exclusion, and total cell numbers were counted by use of a disposable multiwell hemocytometer (Immune Systems, United Kingdom). Airway cells were differentiated by hematoxylin-eosin staining of samples of cells from BAL fluid.

### Flow cytometry.

Prior to staining, cells were blocked with CD16/32 (Fc Block; BD). For surface staining, antibodies against the surface markers CD4, CD8, CD3, CD69, CD11b, CD11c, MHC class II (MHC-II), CD27, and DX5 (BD) were added at a 1:100 dilution for 30 min on ice. Gating for lymphocytes was determined by back gating on CD3/CD8 double-positive cells. For the detection of intracellular cytokines, cells were incubated with 50 ng/ml phorbol myristate acetate, 500 ng/ml ionomycin, and 10 μg/ml brefeldin A for 4 h at 37°C. Samples were permeabilized with 0.5% saponin in phosphate-buffered saline (PBS) for 10 min. Anticytokine antibodies (anti-IFN-γ, anti-IL-4; BD) or isotype controls were added for a further 20 min at room temperature. Cells were analyzed on a CyAn ADP flow cytometer (Dako), with data on at least 50,000 events being collected.

### Inflammatory mediators.

**FIG 1:**
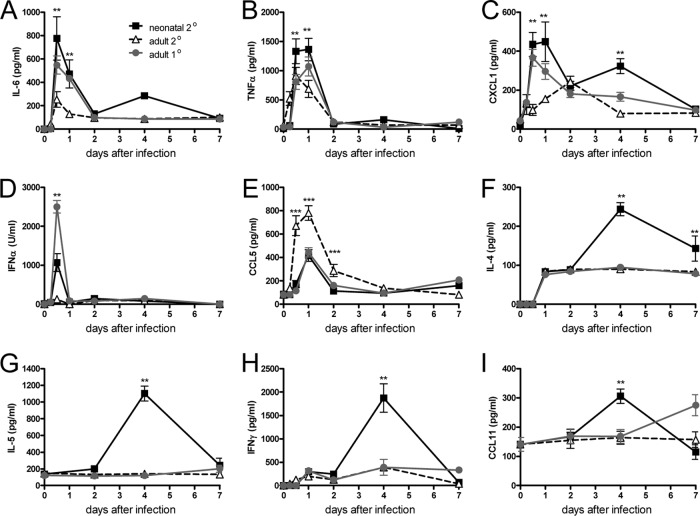
Neonatal RSV infection leads to enhanced chemokine and proinflammatory cytokine release on subsequent RSV reinfection. BALB/c mice were primed with 4 × 10^4^ FFU/g body weight of RSV A2 intranasally at 4 days or 4 weeks of age or left naive. At 8 weeks of age, all mice were infected with 10^6^ FFU of RSV. BAL was carried out at various time intervals after infection, and the levels of the following cytokines and chemokines were determined: IL-6 (A), TNF-α (B), CXCL1 (C), IFN-α (D), CCL5 (E), IL-4 (F), IL-5 (G), IFN-γ (H), and CCL11 (I). Data are from 1 experiment, and points represent the mean ± SEM of 4 mice per group per time point. **, *P* < 0.01, comparing neonates with secondary infection with adults with secondary infection; ***, *P* < 0.001, comparing neonates with secondary infection with adults with secondary infection.

The levels of inflammatory mediators in the BAL fluid were measured. In the time course experiment (depicted in Fig. 1), IL-4, IL-5, IL-6, tumor necrosis factor alpha (TNF-α), CXCL1, CCL5, and IFN-γ were measured by use of a Luminex kit (Millipore), IFN-α was measured by ELISA using capture and detection antibodies (PBL; Interferon Source), and CCL11 was measured by use of an ELISA DuoSet system (R&D Systems). In subsequent studies, IFN-γ and IL-4 were measured by use of the ELISA DuoSet system (R&D Systems). The limits of detection were as specified by the manufacturer.

### Statistical analysis.

Results are expressed as the mean ± standard error of the mean (SEM); statistical significance was calculated by analysis of variance, followed by Tukey tests when there were 3 groups and *t* tests for the comparison of 2 groups, using GraphPad Prism software.

## RESULTS

### Neonatal RSV infection leads to enhanced production of airway inflammatory mediators on reinfection.

We have previously observed that secondary infection of mice initially infected as neonates led to an enhanced inflammatory response on reinfection with equal gaps either between infections or at the same age on reinfection (5–7). We wished to determine which inflammatory mediators were associated with this response. Mice were initially infected as neonates (4 days of age) or adults prior to secondary RSV reinfection; primary adult infection was used as a control. In the early stages of secondary RSV reinfection, mice first infected as neonates had significantly higher levels of mediators associated with innate cell recruitment and activation than mice first infected as adults (Fig. 1A to E). Mice that had initially been infected as neonates had significantly higher levels of IL-6 (*P* < 0.01; Fig. 1A), TNF-α (*P* < 0.01; Fig. 1B), and CXCL1/keratinocyte chemoattractant (*P* < 0.01; Fig. 1C) at 12 h and 24 h after reinfection than mice first infected as adults. The levels of IL-6, TNF-α, and CXCL1 after reinfection of neonatally infected mice were similar to those after primary adult RSV infection. Neonatally infected mice also had an acute peak of IFN-α production at 12 h after reinfection that was significantly greater than that after adult primary and secondary infection (*P* < 0.05; Fig. 1D). There was significantly more CCL5/RANTES after adult secondary infection than after neonatal secondary infection (*P* < 0.01; Fig. 1E).

At later stages of the secondary infection, there were increased levels of cytokines indicative of recruitment and activation of T cells (Fig. 1F to I). As observed previously (5), neonatally infected mice produced significantly higher levels of IL-4 (*P* < 0.01; Fig. 1F), IL-5 (*P* < 0.01; Fig. 1G), IFN-γ (*P* < 0.01; Fig. 1H), and CCL11/eotaxin (*P* < 0.01; Fig. 1I) on day 4 after reinfection and IL-4 (*P* < 0.01) on day 7 after reinfection, though it should be noted that the baseline levels of IL-5 and CCL11 were high.

### Neonatal RSV infection leads to increased innate effector cell recruitment on subsequent RSV reexposure.

**FIG 2:**
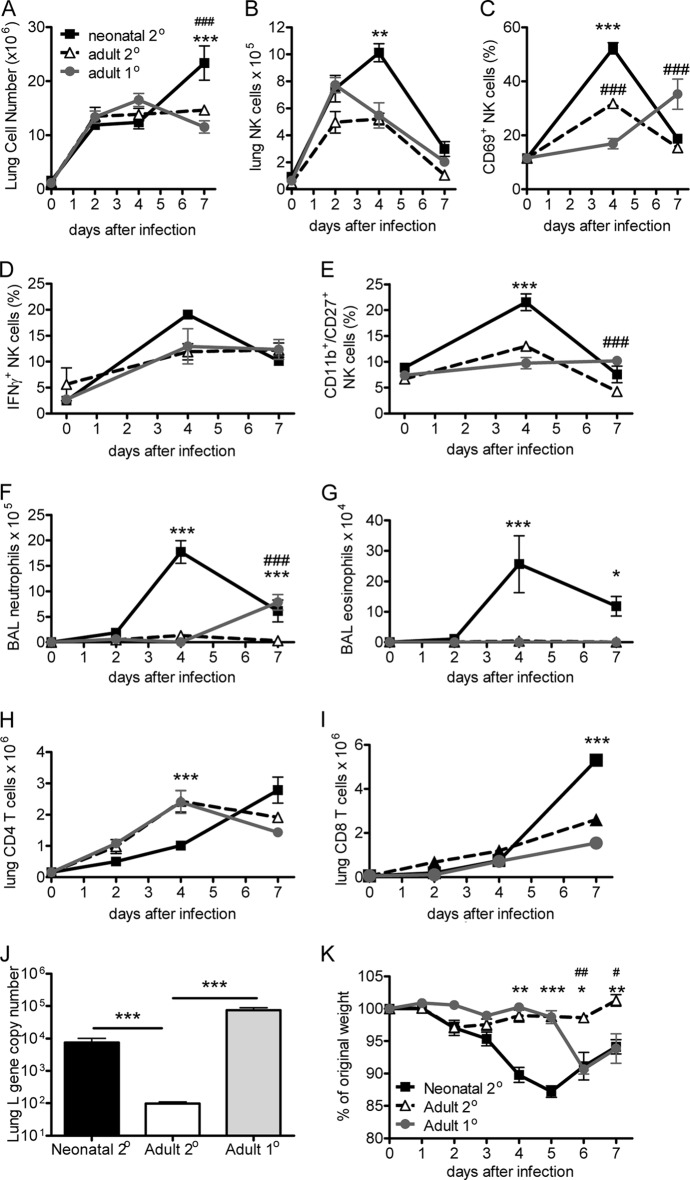
Neonatal RSV infection leads to disease and increased innate effector cell recruitment on subsequent RSV reinfection. BALB/c mice were initially infected with 4 × 10^4^ FFU/g body weight of RSV A2 intranasally at 4 days or 4 weeks of age or left naive. At 8 weeks of age, all mice were infected with 10^6^ FFU of RSV A2. (A) Total lung cell numbers. The proportions of lung NK cells (CD3-DX5^+^; B) that were active (CD69^+^; C), IFN-γ producing (D), or CD27^+^ (E) and the proportions of CD4 T cells (H) and CD8 T cells (I) were determined by flow cytometry. (F, G) BAL fluid neutrophils (F) and eosinophils (G). (J) Lung viral load on day 4 after infection. (K) Weight was monitored following infection. Data are representative of 3 independent repeats, and points or bars represent the mean ± SEM of 5 mice per group per time point. *, *P* < 0.05 between neonates with secondary infection and adults with secondary infection; **, *P* < 0.01 between neonates with secondary infection and adults with secondary infection; ***, *P* < 0.001 between neonates with secondary infection and adults with secondary infection; #, *P* < 0.05 between adults with primary infection and adults with secondary infection; ##, *P* < 0.01 between adults with primary infection and adults with secondary infection; ###, *P* < 0.001 between adults with primary infection and adults with secondary infection.

We then investigated whether the altered profile of airway inflammatory mediators affected cell recruitment. We measured cell recruitment to the lungs and airways on days 0, 2, 4, and 7 of RSV reinfection. In all groups, there was an increase in the total number of cells recruited to the lungs, with significantly more cells being found on day 7 after reinfection of neonatally infected mice than adult infected mice (*P* < 0.01; Fig. 2A). There was also an influx of NK cells to the lungs on day 2 after RSV reinfection, regardless of previous exposure (Fig. 2B). In primary or secondary adult infection, this influx remained at the same level on day 4 after infection and then returned to baseline levels. However, neonatally infected mice had a significant increase in NK cells on day 4 after reinfection, coinciding with the peak of weight loss (*P* < 0.01). There were more CD69^+^ (*P* < 0.001; Fig. 2C) and IFN-γ-expressing (Fig. 2D) NK cells in neonatally infected mice at day 4 after reinfection. CD27^+^ NK cells have greater effector function, a lower activation threshold, and a higher responsiveness to chemokines (12). We found that prior to reinfection the vast majority of lung NK cells were CD11b^+^ CD27^−^, irrespective of the age at initial exposure to RSV. By day 4 after infection, 21.5% (±1.6%) of the NK cells in the mice infected as neonates but only 13% (±0.4%) of the NK cells in adult infected mice were CD11b^+^ CD27^+^ (*P* < 0.01; Fig. 2E). There were also significantly more neutrophils (*P* < 0.001; Fig. 2F) and eosinophils (*P* < 0.05; Fig. 2G) in the airways of mice that had initially been infected with RSV as neonates, with levels peaking on day 4.

Reflecting the cytokine profile, primary neonatal infection altered the T cell response to reinfection. Mice previously infected as neonates had a peak influx of both CD4 and CD8 T cells at day 7 after infection. Mice previously infected as adults had a peak of CD4 T cells at day 4, and mice infected as neonates had a peak of CD4 T cells at day 7 (Fig. 2H). There were significantly more CD8 T cells recruited to the lungs of neonatally infected mice on day 7 after reinfection (*P* < 0.001; Fig. 2I). As seen before (5), there were also more IL-4-producing CD4 cells and IFN-γ-producing CD8 T cells in neonatally primed mice than adults, peaking at day 7 (data not depicted). In spite of the increased inflammation, mice initially infected as neonates had a greater viral load than mice initially infected as adults (Fig. 2J). As previously observed, age at initial RSV infection affected the weight loss profile during secondary reinfection. In mice initially infected as neonates, reinfection caused weight loss starting on day 2 and peaking on day 5, while primary adult infection was protective against weight loss during reinfection (Fig. 2K). These data suggest that innate cell recruitment strongly correlated with disease, as measured by the amplitude of cytokine production, cellularity, and weight loss.

### Natural killer cells contribute to the disease seen following RSV reinfection.

**FIG 3:**
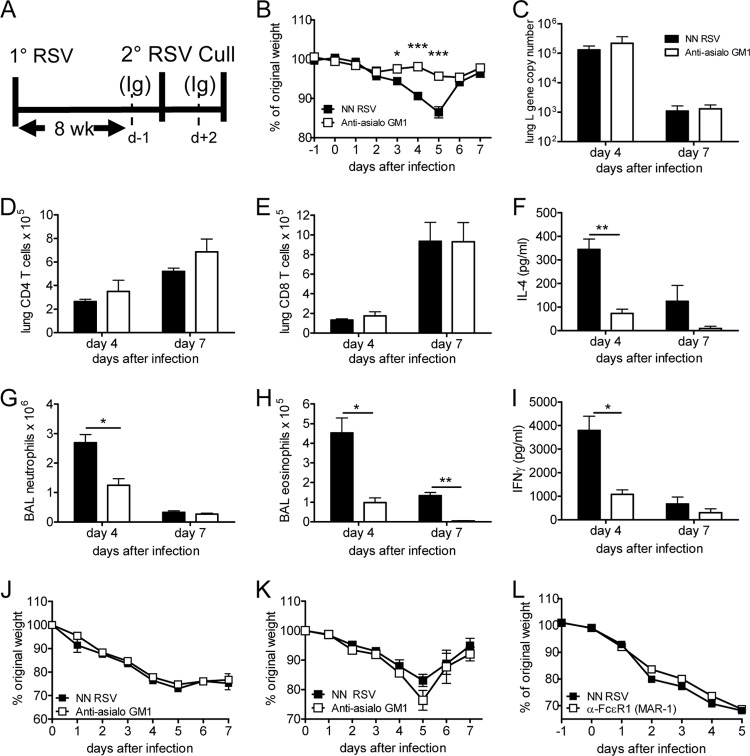
Disease during reinfection following primary neonatal (NN) RSV infection requires NK cells. Four-day-old BALB/c mice were infected i.n. with 10^5^ FFU RSV; 8 weeks later, the mice were reinfected with 10^6^ FFU of RSV. On days −1 and +2 after reinfection, mice were treated i.v. with anti-asialo-GM1 or control serum. (A) Schematic of depletion (d, day). (B) Weight was monitored following reinfection. Viral load (C) and CD4 cell (D) and CD8 cell (E) numbers in the lung and neutrophil numbers (G) eosinophil numbers (H), IFN-γ levels (I), and IL-4 levels (F) in the BAL fluid were measured on days 4 and 7 after infection. Mice were infected with RSV as neonates and treated with anti-asialo-GM1 antibodies during primary infection (J) or 4 weeks after primary infection (K) or anti-FcεR1 during secondary reinfection (L), and their weights were measured during secondary reinfection at 8 weeks of age. Points and bars represent the mean ± SEM of 5 mice per group per time point. *, *P* < 0.05; **, *P* < 0.01; ***, *P* < 0.001.

Since the peak of NK cell recruitment and activity coincided with the peak of disease, we wished to determine whether NK cells were causing the weight loss seen. Anti-asialo-GM1 was used to deplete NK cells during secondary reinfection (Fig. 3A) and led to a significant reduction in lung NK cell number (mean lung NK cell numbers for anti-asialo-GM1-treated mice and isotype control mice, 5.7 × 10^4^ and 3.6 × 10^5^, respectively; *P* < 0.001). Compared to the isotype control treatment, anti-asialo-GM1 treatment significantly reduced the weight loss following RSV reinfection (Fig. 3B). NK cell depletion did not alter the viral load (Fig. 3C) or CD4 (Fig. 3D) or CD8 (Fig. 3E) T cell recruitment to the lungs. There was, however, significantly reduced neutrophil (*P* < 0.05; Fig. 3G) and eosinophil (*P* < 0.05; Fig. 3H) recruitment to the airways and significantly less IFN-γ (Fig. 3I) and IL-4 (Fig. 3F) in anti-asialo-GM1-treated mice.

T cell depletion during primary infection reduced disease on secondary infection (6), suggesting that T cells have a role in priming the response or are acting as memory cells. When NK cells were depleted during primary infection, there was no effect on subsequent disease during secondary reinfection (Fig. 3J), nor was there an effect on secondary disease if NK cells were depleted 4 weeks after primary infection and allowed to reconstitute prior to secondary reinfection (Fig. 3K). To control for the off-target effects of anti-asialo-GM1 on basophils (13), we used anti-FcεR1 to deplete basophils during secondary reinfection. Such treatment had no effect on disease (Fig. 3L), suggesting that the effects of anti-asialo-GM1 depletion are via NK cells rather than basophils.

### Macrophages contribute to the disease seen following RSV reinfection.

**FIG 4:**
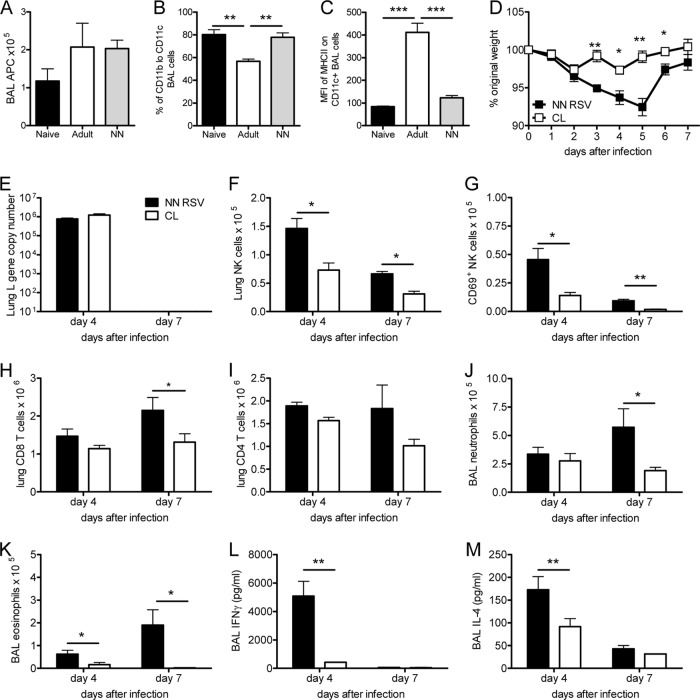
Disease during reinfection following primary neonatal RSV infection requires macrophages. BALB/c mice were initially infected with 4 × 10^4^ FFU/g body weight of RSV A2 intranasally at 4 days or 4 weeks of age. Prior to reinfection at 8 weeks of age, airway APCs were quantified by cytospin analysis (A) and characterized by flow cytometry for dendritic cells and macrophage phenotype (B) and MHC-II expression (C) (MFI, mean fluorescence intensity). In a separate study, 4-day-old BALB/c mice were infected i.n. with 10^5^ FFU RSV; 8 weeks later, the mice were reinfected with 10^6^ FFU of RSV. Three days prior to reinfection, mice were given clodronate-containing liposomes i.n. (open symbols and bars) or PBS (filled symbols and bars). (D) Weight was monitored following reinfection. Lung viral load (E), lung NK cells (CD3^−^ DX5^+^; F) that were active (CD69^+^; G), lung CD8 T cells (H), lung CD4 T cells (I), BAL fluid neutrophils (J) and eosinophils (K), IFN-γ levels (L), and IL-4 levels (M) were determined on days 4 and 7 after infection. Data are representative of 2 independent repeats. Points and bars represent the mean ± SEM of 5 mice per group per time point. *, *P* < 0.05; **, *P* < 0.01; ***, *P* < 0.001.

Since we have previously observed that macrophages play a central role in the recruitment of NK cells following primary RSV infection (14), we wished to determine their role in secondary infection after neonatal infection. We quantified antigen-presenting cell (APC)-like cells by hematoxylin-eosin staining of cells from BAL fluid prior to infection of mice primed when they were adults or neonates or previously naive mice. There was no difference in the total number of APC-like cells in the airways (Fig. 4A). However, when analyzed by flow cytometry, there were significantly fewer CD11b^lo^ CD11c^+^ cells (identified as alveolar macrophages [15]) in the mice primed when they were adults than in the mice primed when they were neonates or naive mice (*P* < 0.01; Fig. 4B) but more CD11b^hi^ CD11c^+^ cells (identified as dendritic cells). As we have previously observed (16), adult infection led to a significant upregulation of MHC-II on CD11c^+^ cells compared to that seen with neonatal infection (Fig. 4C), suggesting that there is a difference in the types of APCs in the airways after adult or neonatal infection.

To test whether the increased numbers of macrophages affected disease, they were depleted in the airways immediately prior to reinfection using intranasal clodronate-containing liposomes (CLs). The dose and route selected have been previously described by us and cause no overt signs of inflammation or disease in the lungs or airways (14). Furthermore, the dose used was also selective for the depletion of MHC-II^lo^ CD11c^+^ cells (macrophages) and not MHC-II^hi^ CD11c^+^ cells (dendritic cells) in the airways (14). CL treatment significantly reduced the macrophage numbers in both the airways (mean macrophage numbers, 5.8 × 10^4^ for treated mice and 1.7 × 10^5^ for untreated mice; *P* < 0.001) and the lung (mean macrophage numbers, 3.5 × 10^5^ for treated mice and 6.7 × 10^6^ for untreated mice; *P* < 0.05) compared to those achieved with treatment with PBS alone. The depletion of macrophages with CLs led to a significant reduction in weight loss on days 3 to 6 of reinfection (*P* < 0.05; Fig. 4D), without altering the viral load or clearance (Fig. 4E). The reduction in weight loss was matched by a significant reduction in the number of NK cells and their activation at both day 4 and day 7 after infection (Fig. 4F and G) and CD8 T cells at day 7 after infection (Fig. 4H), but no change in CD4 T cell recruitment (Fig. 4I). CL treatment led to a significant decrease in neutrophil recruitment on day 7 after infection (Fig. 4J). There were also significantly reduced eosinophil numbers (*P* < 0.05; Fig. 4K), IFN-γ levels (*P* < 0.01; Fig. 4L), and IL-4 levels (*P* < 0.01; Fig. 4M) in the airways on day 4 after infection. Therefore, macrophages play a role in the acute recruitment of other cells to the lungs during RSV reinfection of neonatally infected mice, contributing to disease.

## DISCUSSION

Here we show that neonatal RSV infection primed for pathogenic innate and adaptive immune responses on RSV reinfection in adults. Following reinfection of neonatally infected mice, there was a wave of proinflammatory cytokines followed by an influx of highly activated, proinflammatory NK cells. NK cell depletion reduced disease, suggesting that NK cells are important in promoting pathology in this model. The depletion of macrophages by clodronate liposome treatment during secondary infection significantly reduced cellular recruitment to the lungs and weight loss, indicating that they have a role in the long-term effects of neonatal RSV infection.

Macrophages are a major producer of proinflammatory cytokines in the initial stages of RSV infection (14), and it is likely that they promote inflammation in the lung via their local cytokine and chemokine production. Interestingly, the inflammatory role of macrophages on challenge of neonatally primed mice contrasted with the role of macrophages during secondary challenge of adult primed mice, where they are thought to act in an immunosuppressive capacity (17). Alveolar macrophages from infants have been shown to be immature (18), and the Th2 environment of the neonatal lung may switch macrophages from the classical to the alternatively activated pathway, which is associated with RSV disease (19). The failure to activate macrophages properly may also lead to airway occlusion with cellular debris, contributing to RSV disease (20). We have previously observed differences in antigen-presenting cell populations after adult or neonatal infection and that neonatal treatment with CpG improved the outcome on RSV reinfection associated with a switch to Th1 responses (16).

NK cells play an important role in the defense against lung infection (21). We and others have previously observed that NK cells can contribute to RSV disease (9, 10). It has been proposed that both mouse and human NK subsets are distinguishable through surface levels of CD11b and CD27 (12). CD27^+^ NK cells display greater effector function, a lower activation threshold, and a higher responsiveness to chemokines (12). Importantly, these two subsets also display distinct localizations, with CD27^+^ NK cells being restricted to the systemic/lymphoid compartment, while usually only CD27^−^ NK cells are found in the periphery (12). On day 4 after infection, there was a rapid accumulation of CD11b^+^ CD27^+^ NK cells and even some CD11b^−^ CD27^+^ NK cells in the mice primed as neonates, a feature not seen in either naive mice or mice primed as adults, where the majority of NK cells remained the expected CD11b^+^ CD27^−^ subset. These activated CD27^+^ NK cells produce high levels of IFN-γ, which may further contribute to the disease seen (10). The role of NK cells was investigated using anti-asialo-GM1 treatment, which does have off-target effects on both basophils (13) and CD8 T cells (22), but in our study, we observed significantly reduced NK cell numbers but not reduced CD8 T cell numbers, as we have observed in other studies (9), and basophil depletion had no effect on disease. These studies suggest that NK cells contribute to the long-term effects of neonatal RSV infection.

Interestingly, in contrast to our findings in secondary RSV infection following neonatal sensitization, the depletion of NK cells during adult RSV infection was reported by others to make no difference to the viral load but to lead to the development of a Th2 response (23). In influenza virus infection, NK cells were implicated in promoting pathology in the lung (24). Thus, NK cells play contrasting roles depending on the immunological context of the infection.

In summary, our study highlights the potential of two components of innate immunity, natural killer cells and alveolar macrophages, to modulate immune responses to infection and dictate the severity of secondary disease. Based on these findings, we propose the following mechanism of the effects of secondary RSV reinfection on neonatally infected mice. Neonatal infection with RSV not only fails to produce a protective antibody response (25) but also primes an inflammatory cellular response. Following viral reinfection, macrophages rapidly produce proinflammatory cytokines, which cause recruitment of inflammatory cells, including NK cells, which in turn further promote pulmonary inflammation. While much attention has been paid to promoting and modulating specific T cell responses to infection, progress in novel treatments for RSV disease are hampered by incomplete understanding of innate and adaptive immunity in early life. Therapeutic targeting of innate immunity may provide a novel, broad-based approach to reducing the severity of not only primary lung infections but also secondary disease; for example, neonatal treatment with CpG improved the outcome on RSV reinfection (16). Further study of the innate response may enrich our understanding of the link between neonatal RSV infection and subsequent wheeze in later childhood.
